# Complete genome sequence of *a* plant associated bacterium *Bacillus amyloliquefaciens* subsp. *plantarum* UCMB5033

**DOI:** 10.4056/sigs.4758653

**Published:** 2014-02-15

**Authors:** Adnan Niazi, Shahid Manzoor, Sarosh Bejai, Johan Meijer, Erik Bongcam-Rudloff

**Affiliations:** 1 Department of Animal Breeding and Genetics, SLU Global Bioinformatics Centre, Swedish University of Agricultural Sciences, Uppsala, Sweden.; 2 Department of Plant Biology and Forest Genetics, Uppsala Biocenter, Swedish University of Agricultural Sciences and Linnéan Center for Plant Biology, Uppsala, Sweden; 3 University of the Punjab, Lahore, Pakistan.

**Keywords:** *Bacillus amyloliquefaciens*, biocontrol, rhizobacteria, priming, stress

## Abstract

*Bacillus amyloliquefaciens subsp. plantarum* UCMB5033 is of special interest for its ability to promote host plant growth through production of stimulating compounds and suppression of soil borne pathogens by synthesizing antibacterial and antifungal metabolites or priming plant defense as induced systemic resistance. The genome of *B. amyloliquefaciens* UCMB5033 comprises a 4,071,167 bp long circular chromosome that consists of 3,912 protein-coding genes, 86 tRNA genes and 10 rRNA operons.

## Introduction

*Bacillus amyloliquefaciens* is a plant-associated species belonging to the family *Bacillaceae*. The members of the genus *Bacillus* are ubiquitous in nature and include biologically and ecologically diverse species, ranging from those beneficial for economically important plants, to pathogenic species that are harmful to humans. *B. amyloliquefaciens* UCMB5033 is a plant growth promoting bacterium (PGPB) that was isolated from a cotton plant [1]. Studies have shown that *B. amyloliquefaciens* UCMB5033 is an important tool for studies of plant-bacteria associations, has potential to confer protection against soil borne pathogens, and to stimulate growth of oilseed rape (*Brassica napus*) [2]. Such traits make UCMB5033 an important tool for studies of plant-bacteria associations and production of compounds that directly or indirectly promote plant growth or stress tolerance. Here we present a description of the complete genome sequencing of *B. amyloliquefaciens* UCMB5033 and its annotation.

## Classification and features

**Figure 1:**
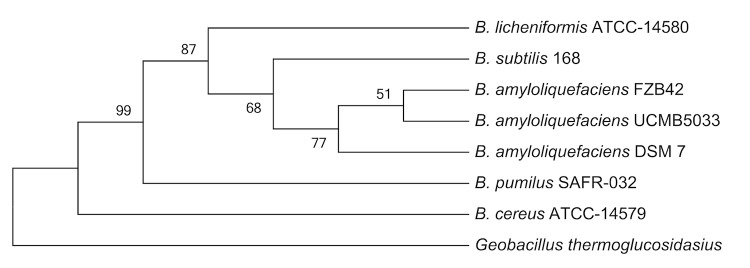
Phylogenetic tree showing the position of *B. amyloliquefaciens* UCMB5033 in relation to other species within the genus *Bacillus*. The tree is based on 16S rRNA gene sequences aligned with MUSCLE [4] was inferred under maximum likelihood criterion using MEGA5 [5] and rooted with *Geobacillus thermoglucosidasius* (a member of the family *Bacillaceae*). The numbers above the branches are support values from 1,000 bootstrap replicates if larger than 50% [6].

Strain UCMB5033 was identified as a member of the *B. amyloliquefaciens* group based on phenotypic analysis [1]. The comparison of 16S rRNA gene sequences with the most recent databases from GenBank using NCBI BLAST [3] under default settings showed that *B. amyloliquefaciens* UCMB5033 shares 99% identity with many *Bacillus* species including *Bacillus atrophaeus* (CP002207.1) and *Bacillus subtilis subsp. spizizenii* str. W23 (CP002183.1). Figure 1 shows the phylogenetic relationship of *B. amyloliquefaciens* UCMB5033 with other species within the genus *Bacillus*. The tree highlights the close relationship of UCMB5033 with the *B. amyloliquefaciens subsp. plantarum* type strain FZB42. The other *B. amyloliquefaciens* type strain DSM 7^T^ representing subsp. *amyloliquefaciens*, displayed less taxonomic relatedness and strain UCMB5033 can thus be regarded as belonging to the subsp. *plantarum* also in line with its plant associated characteristics [7].

### Morphology and physiology

*B. amyloliquefaciens* UCMB5033 is a Gram-positive, rod shaped, motile, spore forming, aerobic, and mesophilic microorganism (Table 1). Strain UCMB5033 is approximately 0.8 µm wide and 2 µm long that can grow on Luria Broth (LB) and potato dextrose agar (PDA) between 20 °C and 37 °C within the pH range 4–8. *B. amyloliquefaciens* UCMB5033 has properties as a plant growth promoting rhizobacterium (PGPR) [2]. The ability to catabolize plant derived compounds, resistance to metals and drugs; root colonization and biosynthesis of metabolites presumably give *B. amyloliquefaciens* UCMB5033 an advantage in developing a symbiotic relationship with plants in competition with other microorganims in the soil microbiota.

**Table 1:** Classification and general features of *B. amyloliquefaciens subsp. plantarum* UCMB5033 according to the MIGS recommendation [8].

**MIGS ID**	**Property**	**Term**	**Evidence code**^a^
	Classification	Domain *Bacteria* Phylum *Firmicutes* Class *Bacilli* Order *Bacillales* Family *Bacillaceae* Genus *Bacillus* Species *Bacillus amyloliquefaciens* Strain UCMB5033	TAS [9] TAS [10-12] TAS [13,14] TAS [15,16] TAS [15,17] TAS [15,18,19] TAS [20-22]
	Gram stain	Positive	IDA
	Cell shape	Rod-shaped	IDA
	Motility	Motile	IDA
	Sporulation	Sporulating	IDA
	Temperature range	Mesophilic	IDA
	Optimum temperature	28**°**C	IDA
	Carbon source	Glucose, fructose, trehalose, mannitol, sucrose, arabinose, raffinose	IDA
	Energy source	--	
	Terminal electron receptor	--	
MIGS-6	Habitat	Soil, Host (Plant)	IDA
MIGS-6.3	Salinity	up to 12% w/v	TAS [20,21]
MIGS-22	Oxygen	Aerobic	IDA
MIGS-15	Biotic relationship	Symbiotic (beneficial)	TAS [2]
MIGS-14	Pathogenicity	None	NAS
MIGS-4	Geographic location	Tajikistan	
MIGS-5	Sample collection time	--	
MIGS-4.1	Latitude	--	
MIGS-4.2	Longitude	--	
MIGS-4.3	Depth	--	
MIGS-4.4	Altitude	--	

a) Evidence codes - IDA: Inferred from Direct Assay; TAS: Traceable Author Statement (i.e., a direct report exists in the literature); NAS: Non-traceable Author Statement (i.e., not directly observed for the living, isolated sample, but based on a generally accepted property for the species, or anecdotal evidence). These evidence codes are from the Gene Ontology project [23].

## Genome assembly and annotation

### Growth conditions and DNA isolation

*B. amyloliquefaciens* UCMB5033 was grown in LB medium at 28°C for 12 hours (cells were in the early stationary phase). The genomic DNA was isolated using a QIAmp DNA mini kit (Qiagen).

### Genome sequencing

*B. amyloliquefaciens* UCMB5033, originally isolated from cotton plant, was selected for sequencing on the basis of its ability to promote rapeseed growth and inhibit soil borne pathogens. Genome sequencing of *B. amyloliquefaciens* UCMB5033 using Illumina multiplex technology and Ion Torrent PGM systems was performed by Science for Life Laboratory (SciLifeLab) at Uppsala University. The genome project is deposited in the **Genomes On Line Databases** [24] and the complete genome sequence is deposited in the ENA database under accession number HG328253. A summary of the project information is shown in Table 2 and its association with MIGS identifiers.

**Table 2:** Genome sequencing Project information

**MIGS ID**	**Property**	**Term**
MIGS-31	Finishing quality	Finished
MIGS-28	Libraries used	Illumina PE (75bp reads, insert size of 230bp), IonTorrent single end reads
MIGS-29	Sequencing platforms	Illumina GAii, IonTorrent PGM Systems
MIGS-31.2	Fold coverage	140× Illumina; 35× IonTorrent
MIGS-30	Assemblers	MIRA 3.4 and Newbler 2.8
MIGS-32	Gene calling method	PRODIGAL, AMIGene
	ENA Project ID	PRJEB3961
	Date of Release	September 8, 2013
	INSDC ID	HG328253
	GOLD ID	Gc0053646
	Project relevance	Biocontrol, Agriculture

### Genome assembly

The genome of *B. amyloliquefaciens* UCMB5033 was assembled using 21,919,534 Illumina paired-end reads (75bp) and 1,922,725 single-end reads (Ion Torrent). The chromosome of size 4,071,167 bp was assembled by providing paired-end reads to MIRA v.3.4 [25] for reference-guided assembly using the available genome sequence of *B. amyloliquefaciens* UCMB5036 (accession no. HF563562) [26]. Whereas, single-end reads were assembled with Newbler v.2.8 by a *de novo* assembly method. Both forms of assemblies were compared after alignment to identify indels and cover gap regions using Mauve genome alignment software [27].

### Genome annotation

The genome sequence was annotated using a combination of several annotation tools *via* the Magnifying Genome (MaGe) Annotation Platform [28]. Genes were identified using Prodigal [29] and AMIGene [30] as part of the MaGe genome annotation pipeline followed by manual curation. Putative functional annotation of the predicted protein coding genes was done automatically by MaGe after BlastP similarity searches against the Uniprot and Trembl, TIGR-Fam, Pfam, PRIAM, COG and InterPro databases. The tRNAScanSE tool [31] was used to find tRNA genes. Ribosomal RNA genes were identified using RNAmmer tool [32].

## Genome properties

**Figure 2:**
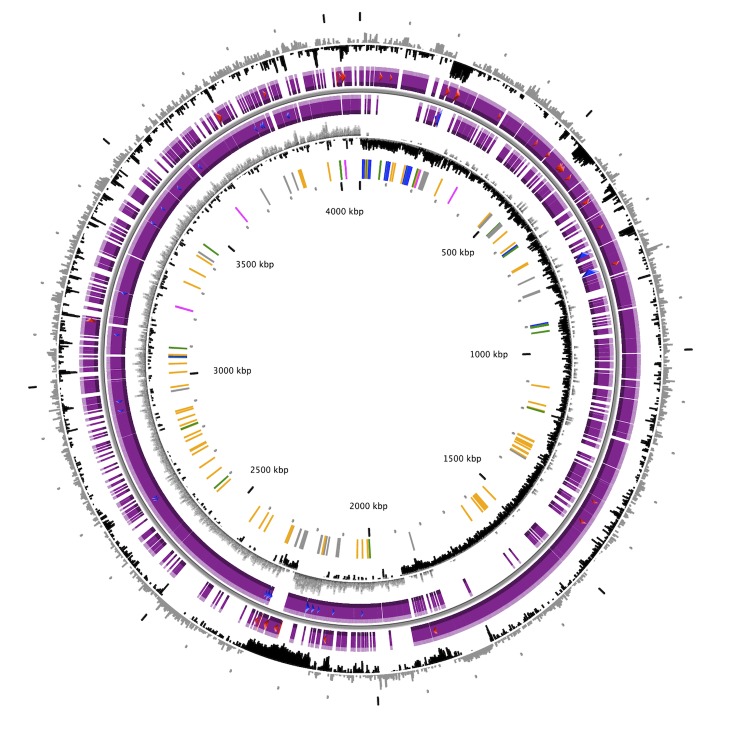
Graphical circular map of the *B. amyloliquefaciens* UCMB5033 genome. From outer to inner circle: (1) GC percent deviation (GC window - mean GC) in a 1000-bp window. (2) Predicted CDSs transcribed in the clockwise direction. (3) Predicted CDSs transcribed in the counter-clockwise direction. Red and blue genes displayed in (2) and (3) are MaGe validated annotations and automatic annotations, respectively. (4) GC skew (G+C/G-C) in a 1,000-bp window. (5) rRNA (blue), tRNA (green), non-coding_RNA (orange), Transposable elements (pink) and pseudogenes (grey).

The *B. amyloliquefaciens* UCMB5033 genome consists of a circular chromosome of size 4,071,168 bp. The genome having G+C content of 46.19% were predicted to contain 4,095 predicted ORFs including 10 copies each of 16S, 23S, and 5S rRNA; 86 tRNA genes, and 3,912 protein-coding sequences with the coding density of 87.51% (Figure 2). The majority of protein coding genes (81%) was assigned putative functions while those remaining were annotated as hypothetical or conserved hypothetical proteins (Table 3). The distribution into COG functional categories is presented in Table 4.

**Table 3:** Nucleotide content and gene count levels of the genome

Attribute	Value	% of total^a^
Genome size (bp)	4,071,168	100
DNA cding region (bp)	3,565,936	87.5
DNA G+C content (bp)	1,880,879	46.1
Total number of genes^b^	4095	n/a
RNA genes	116	n/a
rRNA operons	10	n/a
Protein-coding genes	3912	100
CDSs with predicted functions	3170	81
Uncharacterized/Hypothetical genes	742	18.1
CDSs assigned to COGs	3506	89.6
CDSs with signal peptides	302	7.7
CDSs with transmembrane helices	1012	25.8

a) The total is based on either the size of the genome in base pairs or the total number of protein coding genes in the annotated genome.

b) Also includes 36 pseudogenes and 66 non-coding RNA.

**Table 4:** Number of genes associated with the 25 general COG functional categories

**Code**	**Value**	**%age**^a^	**Description**
J	159	4.06	Translation
A	1	0.025	RNA processing and modification
K	287	7.33	Transcription
L	141	10.58	Replication, recombination and repair
B	1	0.025	Chromatin structure and dynamics
D	38	0.97	Cell cycle control, mitosis and meiosis
Y	0	0.00	Nuclear structure
V	50	1.27	Defense mechanisms
T	167	4.26	Signal transduction mechanisms
M	196	5.01	Cell wall/membrane biogenesis
N	63	1.61	Cell motility
Z	0	0	Cytoskeleton
W	0	0	Extracellular structures
U	54	1.38	Intracellular trafficking and secretion
O	98	2.5	Posttranslational modification, protein turnover, chaperones
C	181	4.62	Energy production and conversion
G	270	6.9	Carbohydrate transport and metabolism
E	313	8	Amino acid transport and metabolism
F	98	2.5	Nucleotide transport and metabolism
H	145	3.7	Coenzyme transport and metabolism
I	169	4.32	Lipid transport and metabolism
P	167	4.26	Inorganic ion transport and metabolism
Q	163	4.16	Secondary metabolites biosynthesis, transport and catabolism
R	426	10.88	General function prediction only
S	319	8.15	Function unknown
-	406	10.37	Not in COGs

a) The total is based on the total number of protein coding genes in the annotated genome.

## Conclusion

Comparative genome analysis might reveal mechanisms by which UCMB5033 mediates plant protection and growth promotion, will further enable the investigations of the biochemical and regulatory mechanisms behind the symbiotic relationship, and will shed light on the activity of PGPR in different environments.
